# A Sensitive DNAzyme-Based Chiral Sensor for Lead Detection

**DOI:** 10.3390/ma6115038

**Published:** 2013-11-01

**Authors:** Hua Kuang, Honghong Yin, Changrui Xing, Chuanlai Xu

**Affiliations:** State Key Lab of Food Science and Technology, School of Food Science and Technology, Jiangnan University, Wuxi, Jiangsu 214122, China; E-Mails: yin0713@126.com (H.Y.); da_rui12345@163.com (C.X.); xcl@jiangnan.edu.cn (C.X.)

**Keywords:** lead, detection, DNAzyme, circular dichroism (CD), sensor

## Abstract

A DNAzyme-based sensor for the determination and quantification of lead ions (Pb^2+^) has been established, which combines the recognition and catalysis of DNAzyme with the optical properties of nanomaterials. Circular dichroism (CD) signals were obtained by a DNAzyme-based assembly of asymmetric silver nanoparticle (AgNPs) dimers. A good linear relationship between CD signals and Pb^2+^ concentration was obtained ranging from 0.05 ng∙mL^−1^ to 10 ng∙mL^−1^ with a limit of detection (LOD) of 0.02 ng∙mL^−1^. The specificity of this sensor in lead ion detection was excellent, and a satisfactory recovery was obtained in the analysis of tap water samples. The proposed technique possesses both high sensitivity and good specificity, giving it great potential for the analysis of Pb^2+^ in water.

## 1. Introduction

Of the various heavy metal ions found in the environment, lead is a major environmental pollutant and can produce toxic effects in plants and animals due to its non-degradation and persistence in the environment. Human exposure to lead results in renal dysfunction, as well as various neurotoxic symptoms [1,2,3,4]. Traditional methods for lead detection include inductively coupled plasma mass spectrometry (ICP-MS) and atomic absorption/emission spectrometry [5,6]. However, high cost, sophisticated instrumentation and complicated sample pretreatment restrict the wide application of these techniques.

DNAzymes, which belong to functional DNA molecules isolated via *in vitro* selection, have been shown to serve as biocatalysts for their recognition of a large variety of targets in chemical transformations [7]. RNA-cleaving DNAzymes have the merits of simple reaction conditions and fast turnover rates, and have been widely used in the detection of analytes [8,9,10]. DNAzymes, different from protein enzymes, can be synthesized by simple chemical reaction and have been verified as having good thermal stability and activity. As oligonucleotides, DNAzymes can be easily modified using various functional groups and immobilized on solid supports. The integration of DNAzymes with nano-materials has opened an important field in sensor technology, and gives a great advantage for specific molecular recognition and signal transduction based on nano-particles (NPs) [11]. This strategy was first employed by Liu and Lu, who used a metal ions specific DNAzyme for the construction of various types of sensors for selective and sensitive detection [12,13,14,15,16]. Their work included the use of a Pb^2+^-specific DNAzyme known as “8–17” for Pb^2+^ detection [17,18]. Following, the formation of numerous DNAzyme-NPs, sensors were developed using various converted signals such as colorimetric signals, dynamic light scattering (DLS), fluorescence, electrochemical signals, chemiluminescent signals, and surface enhanced Raman scattering (SERS) [17,19,20,21,22,23,24].

Circular dichroism (CD) spectra originating from chiral nanostructures assembled by plasmonic NPs have received more interest recently [25,26,27]. The CD signal of assembled NPs was first reported by Alivisato’s group [28]. There, the CD signal of NPs and nanorods arranged in nanometre-scale helices driven by DNA hybridization were reported [29,30,31,32,33]. Gold nanoparticle dimers and gold nanorod ladder assemblies through immuno-recognition were designed to detect antigens by our group [34,35].

In this work, a Pb^2+^-specific DNAzyme was used in the formation of asymmetric silver nanoparticles (AgNPs). In the absence of Pb^2+^, DNA-modified AgNPs dimers were formed. In contrast, the presence of Pb^2+^ induced the DNAzyme to cleave the substrate strand into two pieces, and the dimers were disassembled. The resulting CD signal from assembled AgNPs was found to have a linear relationship with thePb^2+^ level.

## 2. Results and Discussion

**Figure 1:**
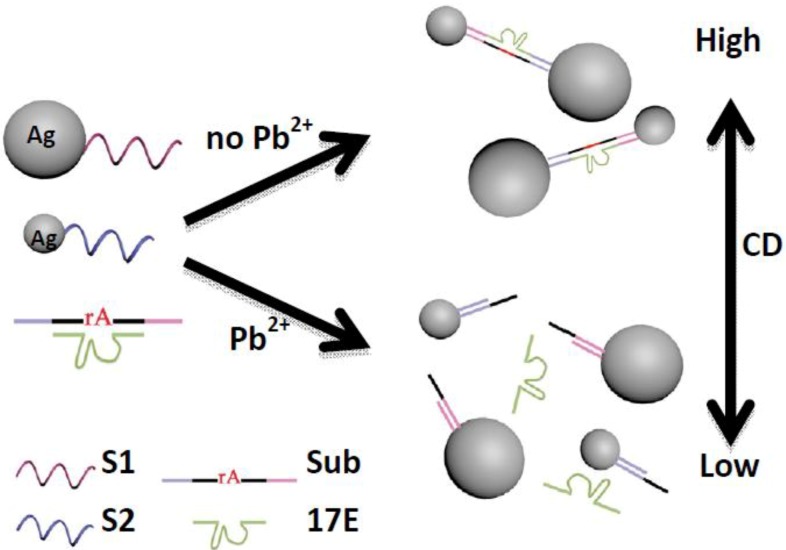
The principle of a DNAzyme-based chirality sensor for Pb^2+^ detection.

The sensing principle for Pb^2+^ detection is illustrated in Figure 1. The DNAzyme-based sensor consisted of four DNA sequences. The DNAzyme known as “8–17” was composed of an enzyme strand (17E) and a substrate strand (Sub). S1 and S2 were coupled with 20 nm and 10 nm AgNPs, respectively. The substrate strand was extended at both ends by 12 bases, which were complementary to the DNA fragments (S1, S2) attached to AgNPs. AgNP dimers were assembled by hybridization of the four DNA fragments, and the CD signal was produced by the asymmetric structure. In the presence of Pb^2+^, the substrate strand was cleaved at the single RNA cleavage site, therefore, the AgNP dimers were disassembled into single NPs by processing at 50 °C.

**Figure 2:**
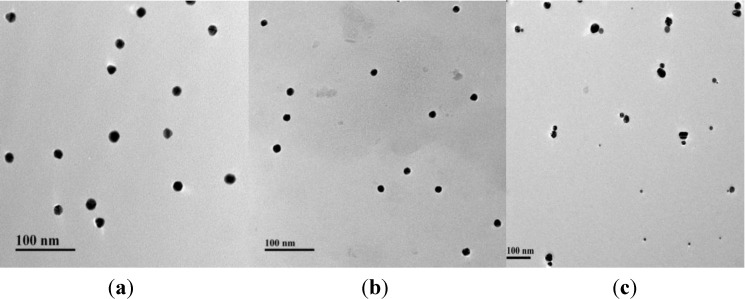
Representative TEM images of AgNPs: (**a**) dispersed single 20 nm AgNPs; (**b**) dispersed single 10 nm AgNPs; and (**c**) a symmetric AgNP dimers.

The TEM images of the two scales of AgNPs showed that they were mono-dispersed (Figure 2a,b), and the UV spectrum of 20 nm AgNPs was larger than that of 10 nm AgNPs (Figure S1). Without Pb^2+^ ions, the asymmetric AgNP dimers were successfully assembled and the hydrodynamic sizes of the assembled dimers were obviously bigger when compared with single NPs (Figure S2). The yield of the dimer assembly was high using the optimized coupling ratio of DNA and AgNPs (10:1). The concentration of Sub and 17E had little influence on dimer assembly. An adequate amount of Sub and 17E were provided for hybridizing with S1 and S2. Using centrifugation, the unreacted DNA was successfully removed (Figure 2c).

**Figure 3:**
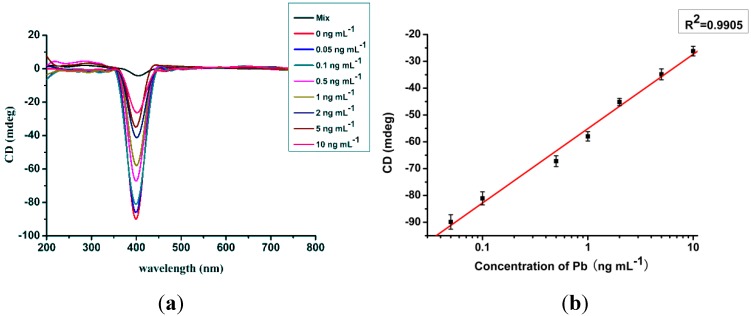
(**a**) CD spectrum under different concentrations of Pb^2+^ (0, 0.05, 0.1, 1, 2, 5, and 10 ng∙mL^−1^); and (**b**) linear relationship for the determination of Pb^2+^.

The sensitivity of the sensor was influenced by the intensity of the CD signals, which were closely related to the number of AgNP dimers. The more AgNP dimers present, the higher the CD signal intensity. Under optimized conditions, the sensitivity of this method was evaluated using different concentrations of Pb^2+^ standard samples (0.05, 0.1, 0.5, 1, 2, 5, and 10 ng∙mL^−1^). The CD intensities are shown in Figure 3a. With an increase in Pb^2+^ concentration, the CD intensity decreased. However, the UV signal showed almost no obvious change, as for the hydrodynamic diameters under different assembly states (Figure S3, Figure S4). The standard curve of Pb^2+^ detection was constructed using the CD intensity and the concentrations of Pb^2+^. A good linear relationship with a correlation coefficient of 0.9905 was observed in the test range from 0.05 ng∙mL^−1^ to 10 ng∙mL^−1^ and the LOD was 0.02 ng∙mL^−1^ (Figure 3b). Kim *et al.* [36] summarized the fluorescent and colorimetric sensors for lead detection in 2012 and the reported sensors have a sensitivity ranging from ng∙mL^−1^ level to mg∙mL^−1^ level. In comparison with the immune-strip measure (LOD value of 2 ng∙mL^−1^), the sensor based on the CD signal is much more sensitive for lead detection [37]. With a unique CD signal, the proposed sensor here manifests excellent sensitivity.

The specificity and selectively of this method was confirmed by Mn^2+^, Zn^2+^, Mg^2+^, Fe^2+^, Ca^2+^, Hg^2+^ and Cu^2+^ at a concentration of 5 ng∙mL^−1^. There were little differences between CD signals from other ions and blank control samples while obvious changes in the CD signal were found in Pb^2+^ detection (Figure S5). The blank tap water samples spiked at various levels (0.05, 0.1, 0.2, 0.5, 1, 2, 5 and 10 ng∙mL^−1^) were detected using this sensor. The recovery ratios were found ranging from 94% to 100% (Table 1). These results indicated that the DNAzyme based sensor was highly specific in the quantitative detection of Pb^2+^.

**Table 1:** Determination of Pb^2+^ in spiked tap water

Spiked level (ng∙mL^−1^)	Detected level Mean ^a^ ± SD ^b^ (ng∙mL^−1^)	Recovery (%)
5	4.81 ± 0.44	96
2	1.97 ± 0.15	98
1	0.94 ± 0.04	94
0.5	0.48 ± 0.05	95
0.2	0.19 ± 0.04	97
0.1	0.10 ± 0.01	100
0.05	0.05 ± 0.01	100

^a^ The mean of five repeats; ^b^ SD = standard deviation.

## 3. Materials and Methods

### 3.1. Materials and Instruments

AgNO_3_, NaBH_4_, polyvinyl pyrrolidone (PVP) and trisodium citrate were all purchased from Sigma-Aldrich (Shanghai, China). Tris-acetate buffer (100 mM) was obtained from Shengon Biotechnology Co. Ltd. (Shanghai, China). Other chemicals were of analytical grade and purchased from J&K Scientific Ltd (Beijing, China). Deionized water was used throughout the experiments and was purified to 18.2 MW (Millipore, Billerica, MA, USA). All the DNA sequences were synthesized by Shengon Biotechnology Co. Ltd. (Shanghai, China) and purified by HPLC. Their detailed sequences were as follows:

S1: 5'-TCACAGATGAGT-SH-3';
S2: 5'-SH-CACGAGTTGACA-3';
17E: 5'-CATCTCTTCTCCGAGCCGGTCGAAATAGTGAGT-3';
Sub: 5'-ACTCATCTGTGAACTCACTAT(rA)GGAAGAGATGTGTCAACTCGTG-3'.

The ultraviolet-visible spectra were measured using an ultraviolet-visible spectrometer (UV, 200–1000 nm) in a quartz cell. The CD spectra were performed on a Bio-Logic MOS-450 CD spectrometer (Grenoble, France). TEM micrographs were collected on a JEOL-2010 microscope (Tokyo, Japan) operated at 120 kV. Dynamic light scattering data were obtained using a Malvern Zetasizer ZS instrument (Malvern, UK) with a 632.8 nm laser source and a backscattering detector at 173°.

### 3.2. Synthesis of Silver NPs

AgNPs scaled with 10 ± 3 nm and 20 ± 3 nm were synthesized according to the method described in the literature with minor modifications [38]. Briefly, 0.6 mL of 0.1 M NaBH_4_ was dissolved in 20 mL ice-cold distilled water, and then 5 mL of a 1% PVP solution as a protecting agent was added. The mixture was continuously stirred in an ice-water bath. Then 5 mL of 1% PVP and 5 mL of 10 mM AgNO_3_ were simultaneously added to the mixture by two constant flow pumps at arate of 30 mL/h. The solution was kept at 80 °C for 3 h to remove excessive NaBH_4_ and then stored at 4 °C until use. Finally, AgNPs with a diameter of 10 nm were obtained. For the preparation of AgNPs with a diameter of 20 nm, similar procedures were used, except 600 μL of a 1% trisodium citrate solution was added in the first step.

### 3.3. AgNPs Functionalized with DNA

The AgNPs (10 nm and 20 nm) were condensed tenfold using centrifugation at 10,000 r/min (for small AgNPs) and 8000 r/min (for larger AgNPs), respectively. The AgNPs were re-suspended in 10 mM Tris-HCl buffer (containing 50 mM NaCl) and the final concentrations of 10 nm and 20 nm AgNPs were 50 nM and 20 nM, respectively. Then, 1 μL of 20 μM S1 was added to 100 μL of 20 nm AgNPs while 1 μL of 50 μM S2 was incubated with 100 μL of 10 nm AgNPs. The coupling process required 12 h and the coupling ratio was 10:1 (DNA-AgNP). The successfully functionalized AgNPs were then centrifuged three times to remove the unreacted DNA and resuspended in 50 μL of 25 mM Tris-acetate (pH 8.2, containing 100 mM NaCl).

### 3.4. Construction of the DNAzyme-Based Sensor

For the construction of the DNAzyme based sensor, 70 μL of 20 nm AgNPs-S1, 30 μL of 10 nm AgNPs-S2, 1 μL of 100 μM Sub and 2 μL of 100 μM 17E were mixed together, and then the sample was heated to 70 °C and allowed to cool slowly to room temperature to promote AgNP dimers assembly. Eight hours later, the AgNP assembly was centrifuged three times to obtain the purified dimers, which were used as sensors for Pb^2+^ detection.

To test the linear response corresponding to the Pb^2+^ amount, 1 μL of Pb^2+^ in different concentrations (final concentrations ranging from 0.05 ng∙mL^−1^ to 10 ng∙mL^−1^) were tested using the system. The reaction system was then incubated in a water bath at 50 °C for 2 min and gradually cooled to room temperature over 2 h in the water bath. The products were analyzed by a CD spectra reader.

### 3.5. Specificity Tests

To evaluate the selectivity of this sensor for Pb^2+^ detection, various other divalent metal ions (Mn^2+^, Zn^2+^, Mg^2+^, Fe^2+^, Ca^2+^, Hg^2+^, Cu^2+^) were tested at a concentration of 5 ng∙mL^−1^. The procedures were the same as the analysis process for Pb^2+^ outlined above. All CD intensity results were compared with the system control and the Pb^2+^ detection system.

### 3.6. Recovery in Tap Water Samples

Pb^2+^ was added to negative tap water samples at the following concentrations 0.05, 0.1, 0.2, 0.5, 1, 2 and 5 ng∙mL^−1^. The recovery ratio was then detected by the DNAzyme-based sensor and was calculated based on the CD signals.

## 4. Conclusions

With the aid of DNAzyme, the degree of AgNP assembly was applied for quantification of Pb^2+^ in water samples. This novel approach takes advantage of the chiral properties of asymmetric AgNP dimers and the unique recognition and catalysis from specific 8-17E DNAzyme. With an increase in Pb^2+^ concentration, the cleavage degree of DNAzyme and the disassembly of the AgNP dimer correspondingly increased, which resulted in the obvious decrease of the intensity of the CD signal. Under optimized conditions, a LOD of 0.02 ng∙mL^−1^ was obtained in the linear range of 0.05–10 ng∙mL^−1^. An excellent specificity to lead analysis was observed in cross-reaction tests. The proposed measure for lead is highly sensitive, simple and specific for lead analysis, which indicates potential application in the future.

## Supplementary Files

Supplementary File 1
