# Supplementary material for: A Sensitive DNAzyme-Based Chiral Sensor for Lead Detection
Source: Materials (Basel). 2013 Nov 1;6(11):5038–46. doi: 10.3390/ma6115038 (PMC5452787; doi:10.3390/ma6115038)

Supplymentary Information

Figure S1. The UV spectrum of 10 nm and 20 nm AgNPs.

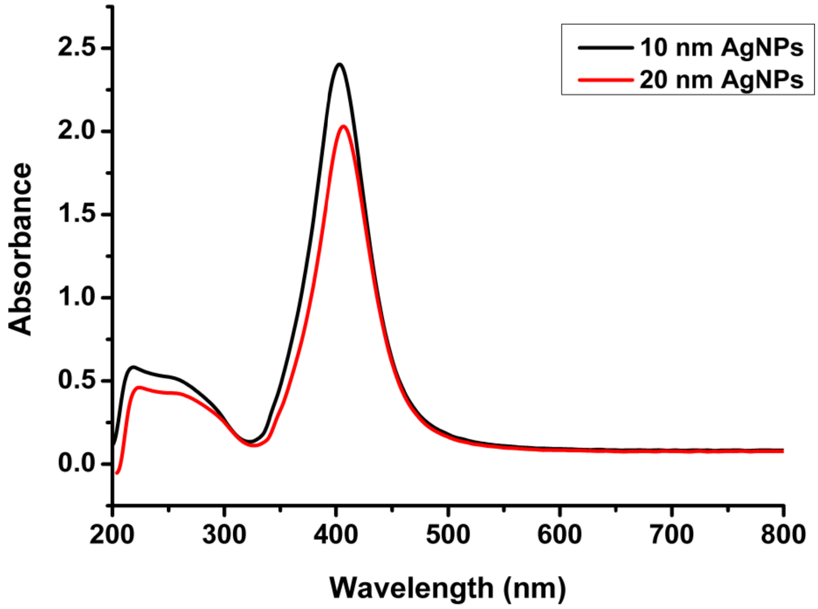

Figure S2. Hydrodynamic diameters of 10 nm AgNPs-S2, 20 nm AgNPs-S1 and asymmetric AgNP dimers

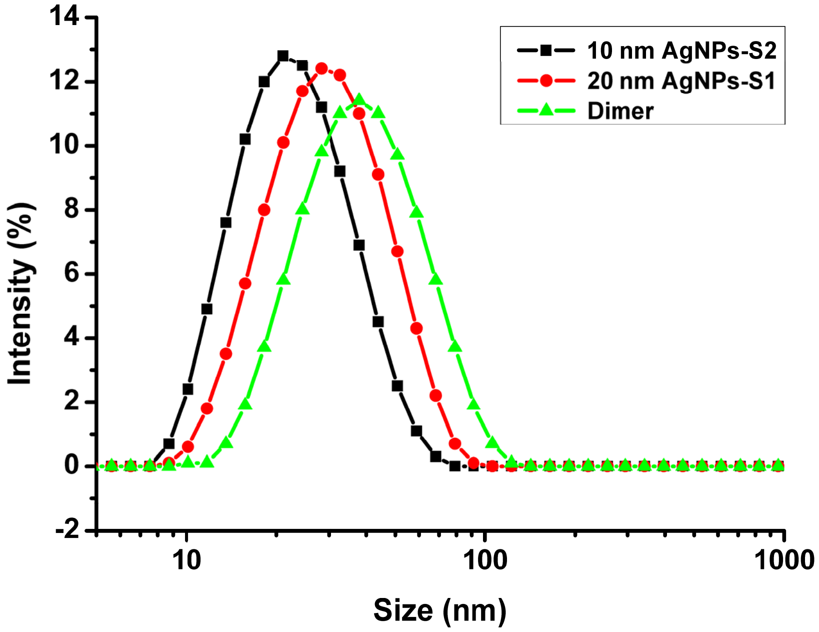

Figure S3. The UV signals under different concentrations of Pb^{2+}(0, 0.05, 0.1, 0.5, 1, 2, 5 and 10 ng·mL^{-1}), and the mix of 10 nm and 20 nm AgNPs without assembly are shown.

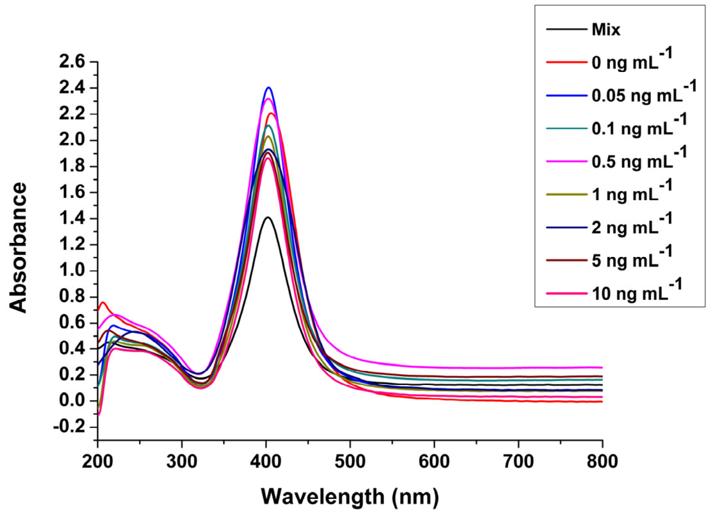

Figure S4. Hydrodynamic diameters at different concentrations of Pb^{2+} (0.05, 0.1, 0.5, 1, 2, 5 and 10 ng·mL^{-1}).

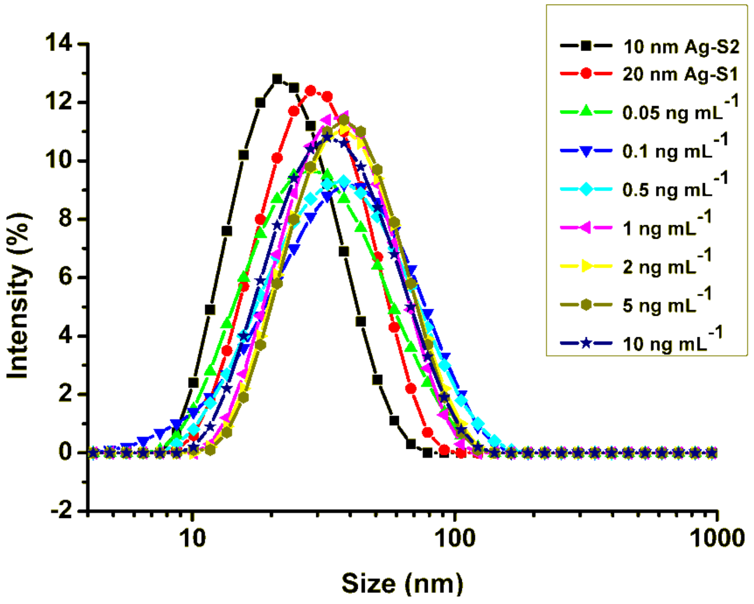

Figure S5. CD spectral change on the determination of Pb^{2+} and other metal ions (Mn^{2+}, Zn^{2+}, Mg^{2+}, Fe^{2+}, Ca^{2+}, Hg^{2+}, Cu^{2+}) at a concentration of 5 ng·mL^{-1}. The control was of AgNP assemblies excluding metal ions.

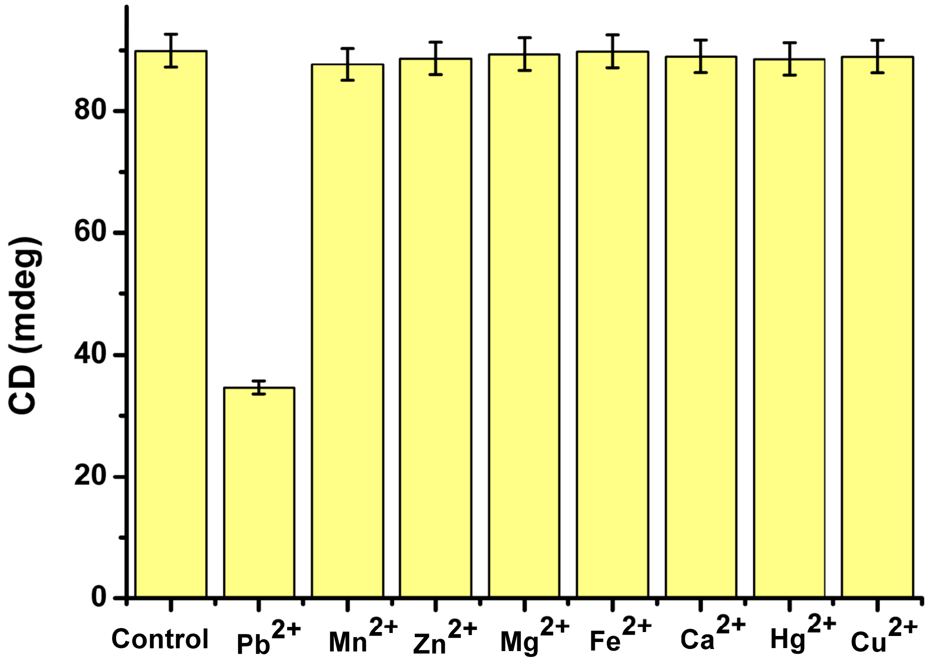

Supplement: Supplementary File 1 [file materials-06-05038-s002.pdf]
